# Myofiber structure, sarcoplasmic reticulum Ca^2+^ handling, and contractile function after muscle‐damaging exercise in humans

**DOI:** 10.14814/phy2.70204

**Published:** 2025-02-02

**Authors:** V. Handegard, P. K. Lunde, M. Frisk, O. Seynnes, N. Ørtenblad, W. E. Louch, G. Paulsen, T. Raastad

**Affiliations:** ^1^ Department of Physical Performance Norwegian School of Sport Sciences Oslo Norway; ^2^ Institute for Experimental Medical Research, Oslo University Hospital and University of Oslo Oslo Norway; ^3^ Department of Sports Science and Clinical Biomechanics University of Southern Denmark Odense Denmark

**Keywords:** Ca^2+^ sensitivity, contractile function, exercise‐induced muscle damage, SR vesicle Ca^2+^ handling, t‐tubules

## Abstract

Exercise‐induced muscle damage (EIMD) is characterized by a severe and prolonged decline in force‐generating capacity. However, the precise cellular mechanisms underlying the observed long‐lasting decline in force‐generating capacity associated with EIMD are still unclear. We investigated *in vivo* force generation and *ex vivo* Ca^2+^‐activated force generation, Ca^2+^ sensitivity, and myofiber Ca^2+^ handling systems (SR and t‐tubules) in human biceps brachii before and 2, 48, and 96 h after eccentrically muscle‐damaging contractions and in non‐exercised control arm. The force‐generating capacity declined by 50 ± 13% 3 h after exercise and was still not recovered after 96 h. The force‐Ca relationship of skinned myofibers revealed an impaired maximal Ca^2+^‐activated force in MHC I‐fibers, but not MHC II‐fibers 48 h after exercise. Further, Ca^2+^ sensitivity was increased in MHC II‐fibers, which was reversed after incubation with a strong reductant. There was a biphasic increase in SERCA sulfonylation, and a parallel reduction in the SR Ca^2+^ uptake rate, with no effects on SR vesicle leak or SR vesicle Ca^2+^ release rate. T‐tubules showed a progressive increase in the density of longitudinal tubules by 96 h after exercise. In conclusion, MHC II‐fiber Ca^2+^ sensitivity was increased 48 h after exercise, attributed to changes in the REDOX status. 96 h after exercise SR vesicle Ca^2+^ uptake was impaired, and an increased number of longitudinal tubules were observed. These alterations may contribute to the impaired force generation evident at the late stage of recovery.

## Graphical abstract

Alterations in myofiber Ca^2+^ handling systems after eccentrically, damaging exercise. Created in https://BioRender.com.

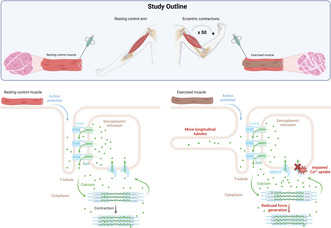

## 1 INTRODUCTION

Exercise‐induced muscle damage (EIMD) is the damage inflicted on myofibers as a result of supramaximal exercise and/or unaccustomed exercise involving both cellular and subcellular disturbances (Clarkson & Hubal, 2002). Previous work has shown that EIMD is characterized by a severe and long‐lasting decline in force‐generating capacity (Hellsten et al., 1997; Jones et al., 1986; Paulsen et al., 2012) accompanied by ultrastructural changes and accumulation of inflammatory cells (Paulsen et al., 2012), and in the most severe cases, myofiber necrosis (Lauritzen et al., 2009). Visible changes in myofiber ultrastructure include sarcomere disruption and Z‐disc “smearing” evident by electron microscopy, and these are hallmarks of EIMD (Hikida et al., 1983; Lauritzen et al., 2009; Raastad et al., 2010; Takekura et al., 2001).

SR Ca^2+^ handling and contractile function are two major determinants of myofiber force production and central to investigate in exercise‐induced myofiber damage. Several studies have focused on this phenomenon by looking at sarcoplasmic reticulum (SR) vesicles containing functional Ca^2+^ channels and pumps, and single‐fiber force‐Ca^2+^ measurements. Single fiber force have been shown to be impaired after fatiguing stimulation in rats with a concomitant increase in Ca^2+^ sensitivity (Watanabe et al., 2015). Impaired (Ingalls et al., 1998; Watanabe et al., 2015) and unaltered (Kamandulis et al., 2017; Nielsen et al., 2007) SR vesicle Ca^2+^ release and uptake have previously been observed, and it remains uncertain how the SR Ca^2+^ handling apparatus is affected. Of note, most studies have been performed in rodents. We therefore highlight the need for human studies investigating SR Ca^2+^ handling and the contractile apparatus after damaging exercise. One possibility is that changes in Ca^2+^ handling following exercise may stem from alterations in the transverse tubular system (t‐system or t‐tubules). These cell membrane extensions span into the skeletal muscle fiber and facilitate action potential (AP) propagation leading to Ca^2+^ release. Changes in the t‐system structure have been reported to occur in rodents after damaging exercise (Takekura et al., 2001; Yeung et al., 2002) and following regular resistance exercise in humans (Cully et al., 2017) with increased presence of both vacuoles and longitudinal tubules. Still, it remains unknown whether alterations in the t‐tubular structure during EIMD are linked to changes in SR Ca^2+^ handling in humans.

It should also be considered that both the induction and the resolution of EIMD centrally involves inflammation (McLoughlin et al., 2003; Round et al., 1987). During EIMD, inflammatory cells migrate to the injured sites to clean up damaged fragments or debris, to regenerate the muscle fiber (Panci & Chazaud, 2021). Activated monocytes and macrophages produce free radicals (Halliwell, 1994). Although necessary for myofiber regeneration, the ROS‐producing inflammatory cells, primarily macrophages, likely increase overall oxidative stress at the injury site (Hellsten et al., 1997), possibly exacerbating tissue damage (Brickson et al., 2003; Zerba et al., 1990). In these processes some proteins are damaged and lose their function whereas others modify their function. Protein function can be regulated by several different post‐translational modifications, including oxidation. Reversible oxidation, such as S‐glutathionylation, can modify protein function and increase function (Adachi et al., 2004), while irreversible oxidation, such as sulfonylation, can exert opposite effects leading to loss of protein function (Pham et al., 2021). Several studies demonstrate that manipulation of ROS can affect muscle fatigue development and recovery, affecting mainly myofibrillar function, with both increases and decreases in myofibrillar Ca^2+^ sensitivity depending on exposure time and concentration (for review see Lamb and Westerblad (2011)). Changes in ROS levels have also been suggested to affect SR Ca^2+^ handling, with reduced Ca^2+^ uptake after prolonged exposure to hydrogen peroxide (H_2_O_2_) (Andrade et al., 1998a) and complete inhibition of Ca^2+^ uptake with exposure to H_2_O_2_ and the ferric iron chelate Fe^3+^‐NTA (Xu et al., 1997). Although it is known that increased ROS generation occurs during exercise with oxidative phosphorylation and after damaging exercise (Brickson et al., 2001; Chiang et al., 2009; Hellsten et al., 1997; Silva et al., 2010, 2011), the extent of ROS production and how it influences SR Ca^2+^ handling after such exercise remains unknown.

This study is an extension of previous work from our lab (Paulsen et al., 2013; Paulsen, Egner, et al., 2010), employing a similar exercise protocol to further investigate the mechanisms causing the severe force decline during EIMD. Specifically, the aim of this study was to link overall contractile function in human muscle to changes in t‐tubular structure, SR Ca^2+^ handling, and Ca^2+^ sensitivity of the contractile apparatus at different stages of exercise‐induced muscle damage (2, 48, and 96 h post‐exercise) in humans. We hypothesize that the decline in force‐generating capacity and the ultrastructural degradation following damaging exercise would be accompanied by disrupted SR Ca^2+^ handling and alterations in t‐tubular structure. Due to the severe decline in maximal force‐generating capacity after damaging exercise reported in several studies, we hypothesized that single fiber force would be impaired, but to a lesser extent than voluntary contraction force.

## 2 MATERIALS AND METHODS

### 2.1 Ethical approval

The study complied to the Helsinki declaration and was approved by the Regional Ethics Committee of Southern Norway (ref. 31,413). The study was registered in clinical trials (no. NCT05036239).

### 2.2 Subjects

Fifteen young men and women (8 ♂, 7 ♀, 26 ± 4 years, 175 ± 9 cm, 75 ± 13 kg; means ± SD) gave informed written consent to participate in the study. We recruited only participants aged between 18 and 35 years. Participants were healthy and did not engage in regular resistance training (<one session per week) specifically targeting the elbow flexor muscles.

### 2.3 Experimental design

To induce muscle damage, participants performed 50 maximal, unilateral eccentric contractions of the elbow flexors while seated in a preacher curl bench with their wrist fixated in a hand cast attached to a dynamometer (Humac NORM, CSMi, Stoughton, MA, USA). The fixation of the wrist was employed in order to minimize the contribution from wrist flexors. The damaging exercise was randomized to either the dominant or non‐dominant arm, and the contralateral arm served as control. The exercise was separated into 10 sets of five repetitions with 30 s of rest between sets. The elbow joint was extended isokinetically at a velocity of 30°/s. Participants were encouraged to withhold each extension (repetition) with maximal effort.

### 2.4 *In vivo* maximal force generating‐capacity and peripheral low‐frequency fatigue

To investigate maximal force‐generating capacity of the elbow flexors, maximal voluntary isometric contraction (MVIC) was assessed at 80° elbow flexion (0° = full extension) using a Humac NORM dynamometer (CSMi, Stoughton, MA, USA). Participants were seated in the same position as during the damaging exercise. Maximal voluntary isometric contraction was assessed in both the exercised and control arms prior to the exercise and 5 min, 3, 24, 48, 72, and 96 h after exercise. Peripheral low‐frequency fatigue was assessed using electrical stimulation (Digitimer; DS7AH, Hertfordshire, UK) of the elbow flexors at 20 and 50 Hz stimulation with a train duration of 400 ms and with the use of skin electrodes; whereby a decrease in the ratio between the force output at 20 and 50 Hz represents low‐frequency fatigue. This assessment was performed prior to and 1 h after exercise. The electrodes were reinforced with extra tape to ensure they remained in place for the duration of the assessment, reducing variation that would arise from different electrode placement in pre‐ and post‐measurements.

Torque during MVIC and electrical stimulation was recorded using a Biopac MP150 (Biopac Systems Inc., Goleta, California, USA) and analysed with a custom‐made Python script in Spyder (version 3.8.8, Python Software Foundation). Peak torque was extracted from the recordings. All measurements were gravity corrected.

For the 20/50 Hz ratio measurements, it was necessary to exclude measurements from eight participants due to artefactual deformations of the torque signal. In such cases the torque level was extremely low and biphasic, with negative values. The reason for this phenomenon is unclear and may be linked to the extreme exhaustion of some participants. Alternatively, the radial nerve innervating the m. triceps brachii may have been stimulated by the electrical current, which could lead to extension of the elbow joint and “negative” force development.

### 2.5 Biopsy procedure

Biopsies were collected 2, 48, and 96 h after completion of the damaging exercise from both the exercised and control arms. A modified Bergström technique was used to obtain biopsies. In brief, a 6 mm‐Pelomi needle was inserted into the midsection of biceps brachii, and tissue samples were obtained using manual suction. The skin area around the incision and the adjoining part of the fascia (epimysium) were anesthetized (Xylocain adrenaline, 10 mg/mL + 5 μg/mL, AstraZeneca, London, UK). Participants laid in a supine position during the procedure. Repeated biopsy insertions were placed 1–2 cm medially or laterally from the previous insertion. Samples for single fiber analyses required 1–2 cm long, intact bundles of fibers. Fiber bundles employed for immediate analysis (<4 h) of t‐tubular structure were submersed in ice‐cold paraffin oil and placed on ice. Fiber bundles used for analysis of single fiber contractile function were placed in a 50% glycerol buffer (5 mM EGTA, 2 mM ATP, 2 mM MgCl_2_, 150 mM K‐proprionate, pH 7.1) in 4°C overnight, and subsequently moved to −20°C until analysis. For cryo‐sectioning, each bundle of fibers was placed in OCT compound and frozen in isopentane cooled to −120°C. 20–25 mg of muscle tissue of was blotted on filter paper and manually homogenized with a Potter‐Elvehjem glass homogenizer in homogenization buffer (in mmol/L; 300 Sucrose, 5 NaN_3_, 1 EDTA, 40 L‐Histidin, 40 Tris HCl, and protease inhibitors (Cat.no. #05056489001, Roche, Mannheim, Germany)) for analysis of SR vesicle Ca^2+^ release/content, leak, and uptake. The SR homogenate was snap frozen in liquid nitrogen and stored in −80°C. The remaining part of the sample was rinsed for fat, blood vessels, and connective tissue in saline, and 30–50 mg was frozen in liquid nitrogen for subsequent western blot analysis.

### 2.6 Skeletal muscle tissue analyses

#### 2.6.1 Single fiber T‐tubular structure

Single fibers were dissected in cold paraffin oil under a dissecting microscope using jeweler's forceps. Isolated fibers were rinsed in Tyrode's solution (in mmol/L; 137 NaCl, 5 KCl, 20 NaHCO_3_, 2 MgSO_4_, 1.2 NaH_2_PO_4_, 10 Glucose, pH 7.4) and incubated with 1% CellMask™ Orange (Cat.no. #C10045, Invitrogen, Thermo Fisher Scientific, Waltham, MA, US) in Tyrode's solution for 30 min, after which t‐tubule images were recorded using an LSM800 Airyscan confocal microscope (Zeiss, Jena, Germany) with a 63x magnification oil immersion objective. Images were post‐processed with the Airyscan 2D SR processing function in Zen 2.3 (Zeiss, Jena, Germany). T‐tubule structure was analysed using *Tubulator* (Frisk et al., 2022). In addition, t‐tubule organization was analysed in binarized images with Fast Fourier Transformation (FFT) in MATLAB (Mathworks, Natick, MA, USA), with peak power of the power spectrum employed as a representation of transverse tubules. Finally, manual counting of longitudinal structures (tubules) and blebs was performed to validate *Tubulator*'s ability to distinguish between these structures. In order to be defined as a vacuole, the following criteria had to be fulfilled: (1) fully defined circle, or (2) an extension of the t‐tubule membrane (only visible in very clear images), (3) the circle did not have to exceed the triad in size as long as it was clearly defined, as stated in criteria 1, (4) dots with stronger fluorescence without defined circle was not counted as a vacuole. In order to be counted as a longitudinal tubule, the following criteria had to be fulfilled: (1) longitudinal tubules that were extending from one triad to another, (2) only “thin” tubules, that is, one tubule. Vacuoles can expand across several triads and resemble longitudinal tubules, however, with larger thickness.

The investigator was blinded to which arm that had performed the exercise in all analyses and fully blinded during manual counting.

#### 2.6.2 Single fiber contractile function

Single‐fiber contractile function experiments included fibers from control and exercised arms at 48 h after eccentric contractions from the final five participants included in the study (no. 11–15) to use the freshest tissue samples. A bundle of the stored muscle fibers from each biopsy (∼40 fibers) was blotted and placed in cold paraffin oil (0–5°C). Single muscle fibers were then randomly selected from different sections of the muscle bundle and isolated under a dissecting microscope (Stemi 2000°C, Zeiss, Germany). Fiber CSA was calculated using the average diameter of the fiber in five locations along the length of the fiber and assuming a cylindrical shape of the fiber. After isolation, fibers were mounted between a pin and a force transducer (AME875, Sensonor, Horten, Norway), stretched to 120% of resting length, and immersed in relaxing solution with 10% Triton X‐100 for 3 min to chemically skin the fiber. Fibers were then washed in relaxing solution and subjected to a maximal Ca^2+^ activation to “set” the fiber. Subsequently, fibers were immersed in a relaxing solution followed by solutions with increasing Ca^2+^ concentration, from pCa 6.7 (relaxing solution) to pCa 4.7 (elicits maximal Ca^2+^ activated force). Next, fibers were exposed to the reducing agent *dithiothreitol* (DTT) for 10 min, allowing investigation of fiber oxidization. After DTT exposure, immersion in solutions with increasing Ca^2+^ concentration was repeated. There was no difference in fiber properties between successive recordings of same fiber exposed to repeated step wise Ca^2+^ concentration without DTT.

Intracellular solutions having different concentrations of free Ca^2+^ were made by mixing two different stock solutions as previously published by Stephenson and Williams (1981): (a) a relaxing solution with high EGTA (in mM): 90 HEPES, 10.3 MgO, 50 EGTA, 8Na_2_‐ATP, 10 Na_2_‐CrP and (b) a Ca^2+^ − EGTA solution (in mM): 90 HEPES, 8.1 MgO, 50 EGTA, 48.5 CaCO_3_, 8 Na_2_‐ATP, 10 Na_2_‐CrP. Both solutions had pH 7.10 ± 0.01 and a calculated free [Mg^2+^] of 1 mM (Lamb & Stephenson, 1994). The EGTA/Ca‐EGTA solutions were mixed in appropriate volumes in order to obtain solutions having different [Ca^2+^]_free_: pCa >9.0 (relaxing solution), pCa 7.0, 6.7, 6.4, 6.2, 6.1, 6.0, 5.9, 5.8, 5.7, 5.6, 5.5, and pCa 4.7 (maximal Ca^2+^ activating solution), where pCa = −log [Ca^2+^]. The same stock solutions were used for all single‐fiber force‐pCa analyses. To test if exercise‐induced oxidation exerted an effect on the contractile apparatus, an additional solution was prepared by mixing the EGTA stock solution (see above) with a strong reducing agent [dithiothreitol (DTT)] at 10 mM final concentration from a 1 M stock made in water (Gejl et al., 2016).

##### Single fiber myosin heavy chain isoform assessment

Single fibers from experiments were placed in Eppendorf tubes containing 20 μL sample buffer (10% glycerol, 5% 2‐mercaptoethanol, and 2.3% SDS, 62.5 mM Tris, and 0.2% bromophenol blue at pH 6.8), boiled for 3 min, and stored at −80°C for later determination of myosin heavy chain (MHC) isoform abundance using SDS‐PAGE (Danieli Betto et al., 1986). In brief, fibers were run on an 8% polyacrylamide gel for 42 h and silver‐stained using PlusOne™ Silver Stainingkit (GE Healthcare, Chicago, Illinois, US). MHC I, IIa, and IIx were determined by comparing protein band migration to a standard myosin extract (internal standard based on mixed homogenates) run in the outer and middle lanes of the gel (Hvid et al., 2013; Nielsen et al., 2007). MHC I, IIa, and IIx abundance are presented as a percentage of total MHC. In total, 107 fibers were analysed. Some fibers showed abundance of several isoforms. Fibers expressing both MHC IIa and IIx were classified as MHC II (*n* = 37). Fibers expressing all isoforms or MHC I and IIa but with ≤20% MHC I (*n* = 7) were also classified as MHC II. Fibers with a 40/60 or 50/50 distribution of MHC I and IIa were classified as hybrid fibers (*n* = 2).

#### 2.6.3 SR vesicle assay

SR vesicle Ca^2+^ handling was examined in homogenates with the method of O'Brien (O'Brien, 1990), modified by Li and colleagues (Li et al., 2002). In brief, Ca^2+^ uptake by vesicles was initiated by Na_4_ATP (2.2 mmol/L) and blocked by thapsigargin (1.5 μmol/L; Cat.no. #T9033, Sigma, Schnelldorf, Germany) after 12 min, which elicits a complete and irreversible block of SERCA activity. The Ca^2+^‐filling of vesicles creates a marked concentration gradient which results in Ca^2+^ leakage through the ryanodine receptor. Releasable Ca^2+^, that is, Ca^2+^ content, was estimated by adding 4‐Chloro‐m‐cresol (4‐CmC; 5.5 mmol/L; Cat.no. #24940, Sigma, Schnelldorf, Germany), a Ryanodine receptor opener. The Ca^2+^ leak was calculated between the addition of thapsigargin (SERCA blockage) and 4‐chloro‐m‐cresol (Ryanodine receptor opener). Ca^2+^ fluxes were calculated by the Fura‐2 (Cat.no. #17195, Sigma, Schnelldorf, Germany) 340 nm/390 nm fluorescence ratio with a Hidex Sense Multimodal Microplate Reader (Kem‐En‐Tec Nordic AS, Uppsala, Sweden). The ratio was calibrated to [Ca^2+^] by the following equation: Kd*((R‐Rmin)/(Rmax‐R))*(Sf2/Sb2), where Kd is the dissociation constant of Fura‐2 and Ca^2+^ (224 nmol/L), and R is the 340/390 nm fluorescence ratio. Rmin and Rmax are the fluorescence ratios at very low and saturating [Ca^2+^], respectively. These were obtained by the addition of 3.3 mmol/L EGTA and subsequently 4.8 mmol/L CaCl_2_ to each well at the end of the experiment. Sf2/Sb2 is the ratio of fluorescence at 390 nm when Fura‐2 is Ca^2+^‐free (3.3 mmol/L EGTA) and Ca^2+^‐bound (4.8 mmol/L CaCl_2_).

All recordings were smoothed using TableCurve 2D (Version 5.01, Systat Software Inc.) prior to analysis. The rate constant for Ca^2+^ uptake (k) was calculated by fitting the uptake curve to Ca^2+^ = Ca^2+^0 + ae‐t*k (Sigmaplot, version 14.0, Systat Software Inc). Ca^2+^ leak was calculated as the slope of the Ca^2+^ signal after the addition of the SERCA blocker, Thapsigargin. Leak measurements were normalized to SR Ca^2+^ content and determined as the maximal [Ca^2+^] after the addition of 4‐CmC.

#### 2.6.4 Western blots

Proteins associated with SR Ca^2+^ release (DHPR, RyR), leak (Calstabin), and uptake (SERCA1, SERCA2, and phospholamban) were investigated with Western blotting. In brief, frozen muscle samples were homogenized and fractionated using ProteoExtract® Subcellular Proteome Extraction kit (Cat.no. #539790, Calbiochem, Merck KGaA, Darmstadt, Germany). All proteins were investigated in the membrane fraction. Protein content was measured by RC/DC protein assay (Reagent A, Cat.no. #5000113; Reagent B, Cat.no. #5000114; Reagent S, Cat.no. #5000115; Bovine γ‐Globulin Standard Set, Cat.no. #5000209; all from Bio‐Rad, Hercules, California, US) in fractionated samples. Before gel electrophoresis, 0.13–0.3 μg/μL of protein was loaded to polyacrylamide stain‐free gels (4%–20% polyacrylamide to run all proteins except RyR, Cat.no. #4568094; 7,5% polyacrylamide to run RyR, Cat.no. #4568024; both gels from Bio‐Rad, Hercules, California, US). Each sample was maximized with regards to protein content since sample and antibody testing prior to western blot analyses showed weak signal for some proteins (DHPR, RyR). Further, proteins were separated by gel electrophoresis and transferred to polyvinylidene difluoride (PVDF) membranes. Membranes were blocked by 5% skimmed milk in Tris‐buffered saline with 0.1% Tween 20 (VWR International, Radnor, PA, US) (TBS‐t) for 2 h at room temperature before being incubated with primary antibodies in 1% skimmed milk in 0.1% TBS‐t overnight at 4°C. Secondary antibodies were incubated in 1% skimmed milk in 0.1% TBS‐t for 1 h at room temperature. See Table 1 for specific information about antibodies. Protein bands were visualized using chemiluminescence (SuperSignal™ West Dura Extended Duration Substrate, Cat.no. #34075, Thermo Fisher Scientific, Waltham, MA, US), imaged with the ChemiDoc MP system, and band intensity was quantified using Image Lab (Bio‐Rad, Hercules, California, US). During image acquisition, light exposure time was optimized to each image individually and set to yield an image well below band saturation. Target proteins were normalized to the total protein content by the stain‐free technology developed by Bio‐Rad, accounting for differences in applied protein content. The normalized band intensity for each sample was also normalized to a quality control sample applied on the same gel. The normalization to a quality control sample allow for gel‐to‐gel comparisons (Bass et al., 2017) and, in this study, comparison between different participants. Representative Western blots are presented in Figures 3 and 4.

**TABLE 1:** Overview of antibodies.

Western blot					
Target	Company	Batch (lot)	Dilution	Type	RRID
Calstabin/FKBP12	Abcam; ab2918	*GR32375771*	1:1000	Primary	AB_303413
DHPR/Cav1.1	Invitrogen; MA3‐920	*VB291904*	1:500	Primary	AB_2069575
Phospholamban	Invitrogen; MA3‐922	*T1270655*	1:500	Primary	AB_2252716
Ryanodine Receptor	Abcam; ab2868	*GR3250452*	1:8000	Primary	AB_2183051
SERCA 1	Thermo Fisher; MA3‐912	*SJ257196*	1:2000	Primary	AB_2061281
SERCA 2	Santa Cruz; sc‐376,235	K1918	1:500	Primary	AB_10989947
Anti‐mouse	Thermo Fisher; 31,430	*IK1106742*	1:30000	Secondary	AB_228374
Anti‐rabbit	Thermo Fisher; 31,460	IL 1099247	1:30000	Secondary	AB_228341

Immunohistochemistry				
Target	Company	Dilution	Type	RRID
Dystrophin	DSHB; MANDYS8	1:100	Primary	AB_2618170
Sulfonylated SERCA Cys 674	Bethyl labs	1:500	Primary	
Anti‐rabbit	Alexa‐Fluor 488 IgG H + L	1:200	Secondary	
Anti‐mouse	Alexa Fluor 594 IgG H + L	1:200	Secondary	

#### 2.6.5 Immunohistochemistry

Eight micrometer‐thin sections were cut using a cryostat (CM1860 UV, Leica Microsystems GmbH, Nussloch, Germany), mounted on microscope slides, and air‐dried before being stored at −80°C. All biopsies from one participant (exercised and control samples from 2, 48, and 96 h) were placed on the same microscope slide, enabling within‐participant comparisons. On the day of staining, slides with sections were defrosted, and a PAP pen was used to make a lipid barrier around the sections. Sections were further fixated with Histofix for 5 min and blocked with 10% bovine serum albumin in phosphate buffered saline for 30 min. Primary antibodies against sulfonylated Cys 674 and dystrophin were incubated overnight at 4°C. See Ying et al. (2008) for validation of the sulfonylated Cys 674‐primary antibody. Cysteine 674 exists in both SERCA 1 and 2 (Dremina et al., 2007). Appropriate secondary antibodies were incubated for 1 h at room temperature. See Table 1 for full information about antibodies. Cover slides were mounted using Prolong Gold Antifade with DAPI (Cat.no. #P36935, Invitrogen, Life Technologies, Carlsbad, California, US). An LSM800 Airyscan confocal microscope (Zeiss, Jena, Germany) with a 63x magnification oil immersion objective and Zen 2.3 software with tile scan function were used to obtain images. The image acquisition order was random between all sections on each microscope slide but exposure time was equal for all sections on the same slide. After image acquisition, individual tiles were fused by stitching with a minimal overlap of 5% and max shift of 10%. Staining intensity was measured across at least 100 random fibers in each section, and myosin heavy chain isoform expression was quantified from a neighboring section. In our samples, we measured fiber type distribution to be 51 ± 6% MHC II‐fibers. A minimum of 50 fibers from both MHC I and MHC II fibers were quantified. The region of interest was manually drawn within each fiber in the dystrophin stain image before intensity was measured in the SERCA sulfonylation stain image. This was done to avoid selection bias when choosing fibers. SERCA sulfonylation staining within myofibers was background subtracted. Fibers along the edge of the sections were not included to avoid possible effects of preanalytical biopsy handling. The investigator was blinded as to whether samples belonged to the damaged or control arm.

#### 2.6.6 Antibodies

The antibodies applied in western blotting and immunohistochemistry are listed in Table 1.

### 2.7 Statistics

Statistical analyses were performed using GraphPad Prism 9.2.0 and SPSS 27. Statistical significance was defined as *p* < 0.05. Data were checked for outliers using the robust regression and outlier removal (ROUT)‐method (*Q* = 1%). The effect of the eccentric exercise was investigated using a two‐way RM ANOVA with time (pre, post 5 min, 2, 3, 24, 48, 72, and 96 h) and condition (exercise and control) as fixed effects, and the Tukey method was employed to correct for multiple post hoc tests. If a variable had missing values, the data were analysed with a mixed model instead. For non‐normally distributed data, a Kruskal–Wallis test with Dunn's multiple comparisons test was used. Single‐fiber Ca^2+^‐elicited force recordings were analysed by curve‐fitting using the non‐linear regression sigmoid curve. In GraphPad Prism, the “dose–response stimulation” was chosen. Variables from force‐pCa recordings were analysed using a mixed model with myosin heavy chain isoform, condition, and antioxidant treatment as fixed effects. To correct for multiple post hoc analyses, a Šídák test was employed. All data are expressed as mean ± standard deviation (SD).

## 3 RESULTS

### 3.1 Fatigue and recovery of force *in vivo*

**FIGURE 1:**
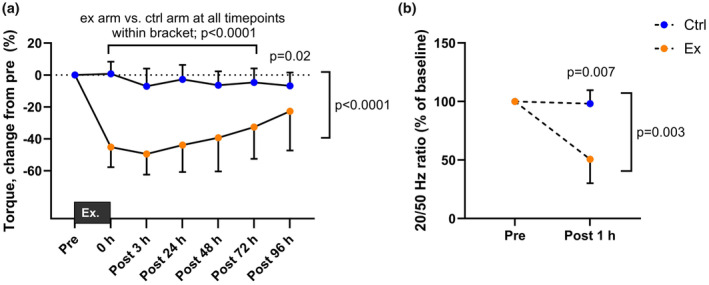
*In vivo* force decline and low‐frequency muscle fatigue. (a) Maximal force‐generating capacity of the elbow flexors before and after the eccentric exercise. Color coding is the same throughout the paper; orange represent exercised arm or muscle tissue samples from the m. biceps brachii of the exercised arm, blue represent resting control arm or muscle tissue samples from the m. biceps brachii of the resting control arm. (b) Low‐frequency fatigue assessed by neuromuscular electrical stimulation the elbow flexors at 20 and 50 Hz. Brackets located on the right side of the figures indicate main and/or interaction effect with *p*‐values. *p*‐values above a certain timepoint indicate significant difference between exercised and control arms at the given timepoint.

Force‐generating capacity, measured as maximal voluntary isometric contraction (MVIC) torque, was markedly reduced following eccentric exercise. Peak impairment of maximal torque was observed 3 h after the completion of exercise (values reduced by 50 ± 13%, *p* < 0.0001), and force‐generating capacity had still not completely recovered at the 96‐h timepoint (*p* = 0.043) in the exercised arm (Figure 1a). Maximal torque in the control arm did not change significantly from baseline at any timepoint (*p* > 0.05). Low‐frequency peripheral muscle fatigue, indicated by a 49 ± 21% reduction in the 20/50 Hz force ratio, was evident for the exercised arm 1 h after eccentric exercise (*p* = 0.002), while no change was observed in the control arm (*p* = 0.969, Figure 1b).

### 3.2 Single fiber contractile function 48 h after eccentric exercise

We next investigated whether the large reduction in maximal force‐generating capacity following eccentric exercise was rooted in altered single‐fiber contractile function. We observed that single fiber specific force (P_0_) was 12% lower 48 h after eccentric exercise in exercised compared to control muscles (Ctrl = 140 ± 67 kN/m^−2^, Ex = 123 ± 58 kN/m^−2^; main effect of exercise, *p* = 0.029). This reduction was driven by a significant decrease in MHC I‐fiber P_0_ in exercised versus control conditions (Table 2, unpaired *t*‐test), which further resulted in an interaction effect between eccentric exercise and MHC isoform (*p* = 0.013).

**TABLE 2:** Single fiber characteristics 48 h after eccentric exercise.

	MHC I fibers				MHC II fibers			
	Untreated condition		DTT‐treated		Untreated condition		DTT‐treated	
	Ctrl	Ex	Ctrl	Ex	Ctrl	Ex	Ctrl	Ex
*n* (fibers)	10	14 (12)	8	14 (12)	33	27	31	27
*F*_max_ (mN)	0.46 ± 0.22	0.48 ± 0.38	0.43 ± 0.19	0.43 ± 0.31	0.69 ± 0.27*	0.61 ± 0.31#	0.59 ± 0.24*	0.52 ± 0.26#
CSA (μm^2^)	3591 ± 1369	4761 ± 1507			5306 ± 2011	4502 ± 2166		
P_0_ (kN/m^−2^)	141 ± 78§	75 ± 32#, §	145 ± 78	71 ± 30	139 ± 65¤	144 ± 55#, *	114 ± 50¤	121 ± 47*
pCa_50_	5.87 ± 0.15	5.99 ± 0.21	5.88 ± 0.16	5.95 ± 0.16	5.83 ± 0.13#, ¤	5.94 ± 0.13*, #	5.78 ± 0.13¤	5.84 ± 0.08*
Hill coef.	−4.05 ± 0.98	−3.53 ± 1.05*	−4.35 ± 1.18	−3.93 ± 1.14	−5.49 ± 1.02#	−5.54 ± 1.69*	−6.29 ± 2.19#	−5.46 ± 2.22

*Note*: “Ex” show results from exercised muscle, “Ctrl” show results from control muscle. F_max_ is the maximal Ca^2+^ activated force, CSA is the cross‐sectional area, P_0_ is the specific force, pCa_50_ is the [Ca^2+^] needed to elicit 50% of max force, that is, the Ca^2+^ sensitivity. Hill coef., that is, Hill coefficient, is the slope of the force‐pCa relationship. #, ¤, *, Similar symbol indicates significant differences (*p* < 0.05) between pairs for each variable separately. §, Similar symbol indicates significant *t*‐test (*p* < 0.05) between the two conditions. Subgroup analysis: results from five participants at 48 h after eccentric exercise.

**FIGURE 2:**
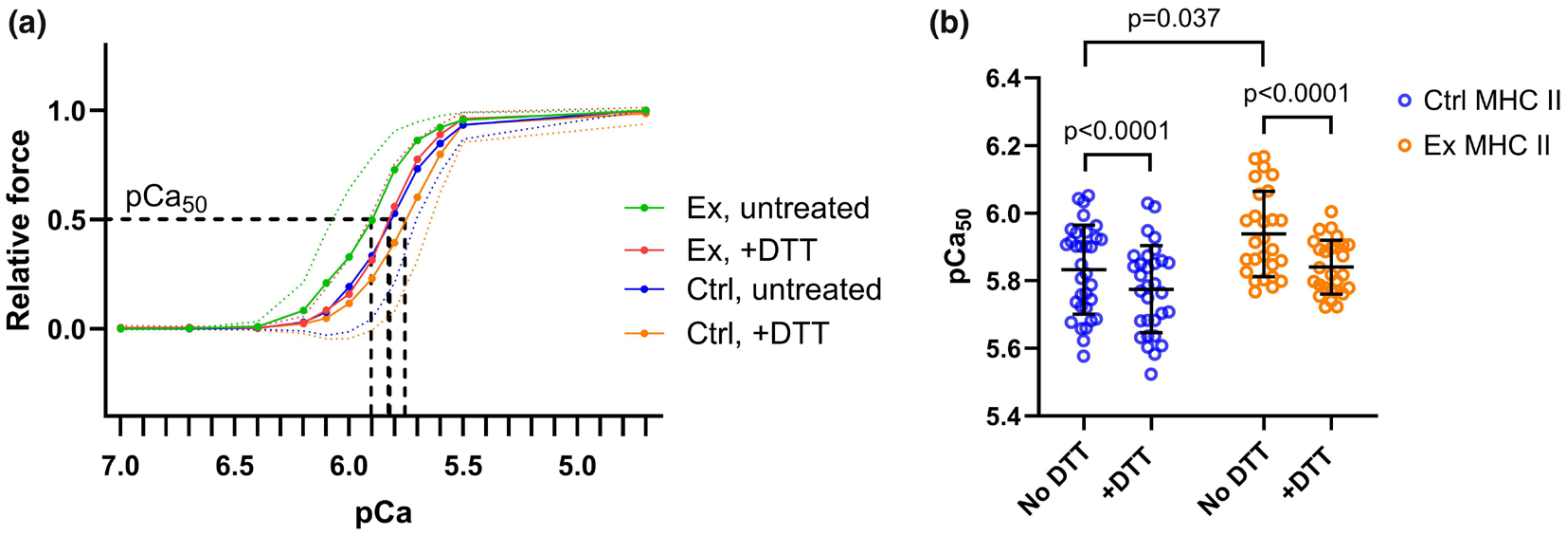
Eccentric exercise and the reducing agent DTT influence single fiber Ca^2+^ sensitivity. (a) Force‐pCa curve showing mean ± SD (dotted lines) of DTT‐untreated exercised myofibers (green), DTT‐treated exercised fibers (red), DTT‐untreated control myofibers (blue), and DTT‐treated control myofibers (orange). All MHC II‐fibers. (b) Ca^2+^ sensitivity (pCa_50_) of MHC II‐fibers. *p*‐values indicate significant differences between myofibers with and without DTT treatment or between myofibers from exercised and control samples.

Interestingly, although specific force was not altered by exercise in MHC II‐fibers, Ca^2+^ sensitivity (pCa_50_) was higher in these fibers compared to controls (Figure 2 and Table 2). No such shift was observed in MHC I‐fibers during exercise. Because increased oxidative stress has been widely reported after eccentric exercise and may critically alter myofilament Ca^2+^ sensitivity, we investigated whether such alterations had contributed to the observed changes in Ca^2+^ sensitivity, by treating the fibers with the reducing agent DTT. This treatment caused a subsequent decrease in Ca^2+^ sensitivity limited to MHC II‐fibers (interaction effect, *p* = 0.0002). Although both control and exercised MHC II‐fibers exhibited a reduction in pCa_50_ following DTT, the decrease was more marked in the exercised fibers (interaction between DTT treatment and exercise, *p* = 0.013). DTT treatment also caused a decrease in P_0_ in MHC II‐fibers only (interaction between DTT treatment and MHC isoform, *p* < 0.0001). The steepness of the force‐pCa relationship, that is, the Hill coefficient, was significantly dependent on MHC isoform (*p* < 0.0001), with greater steepness observed in exercised MHC II‐fibers compared to exercised MHC I‐fibers (*p* < 0.05). Furthermore, DTT treatment also significantly affected the Hill coefficient (main effect, *p* = 0.032), as we observed a significant increase in the force‐pCa relationship steepness in control MHC II‐fibers (*p* < 0.05), while no DTT‐induced increase was observed in exercised MHC II‐fibers. Taken together, these results suggest that MHC II fibers exhibit more oxidization at baseline compared to MHC I fibers, and that eccentric exercise exacerbates this difference leading to increased Ca^2+^ sensitivity of MHC II fibers. Furthermore, exercise per se did not affect the steepness of force‐pCa relationship although both MHC isoform and DTT treatment did. See Table 2 for full results.

### 3.3 SR vesicle Ca^2+^ handling

**FIGURE 3:**
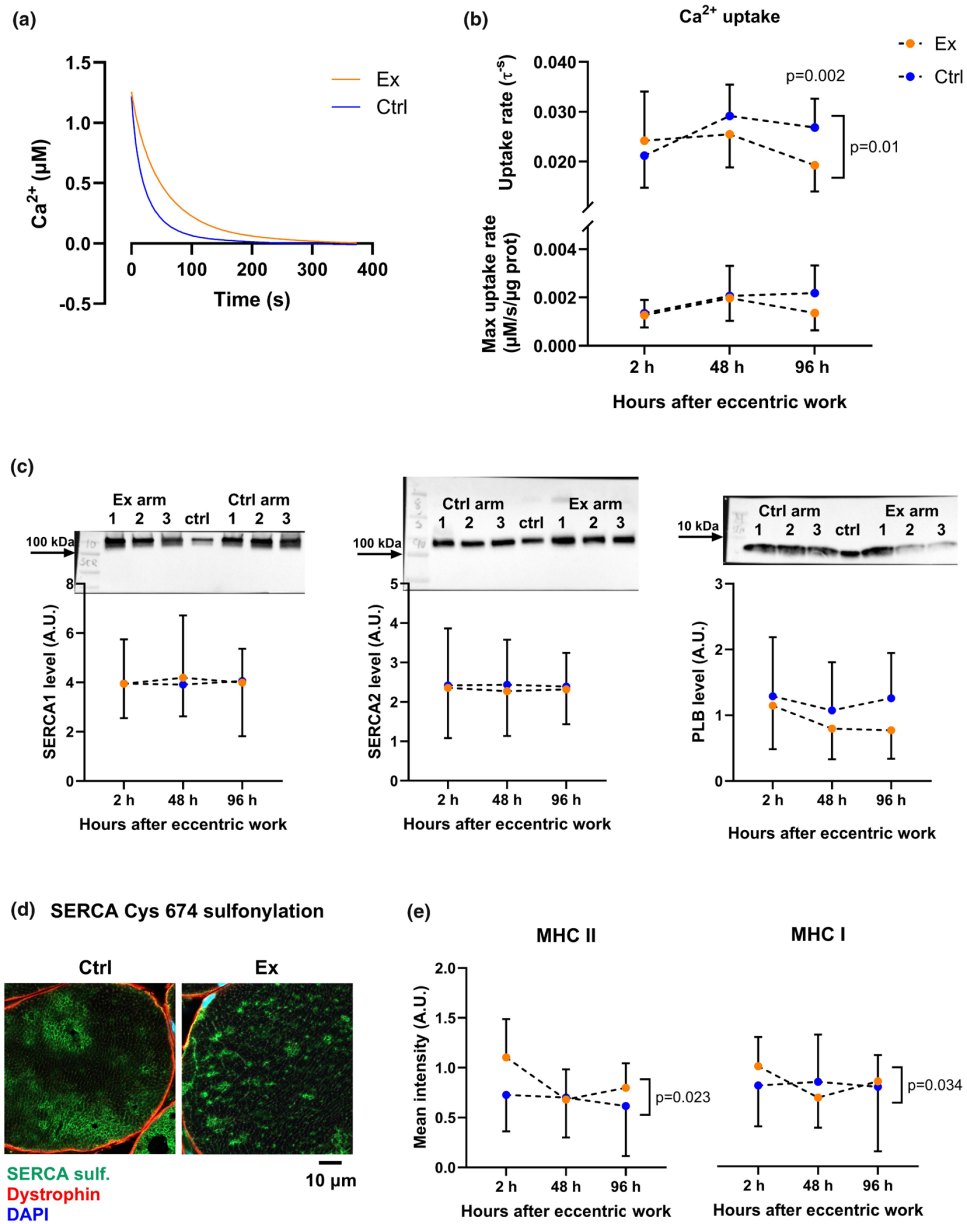
Ca^2+^ uptake rate and possible mechanisms for reduced Ca^2+^ uptake. (a) Representative tracing of Ca^2+^ uptake to SR vesicles. Orange represent exercised arm, blue represent control arm. (b) SR Ca^2+^ uptake rate (maximal and tau). Brackets with *p*‐values located on the right side of a figure indicates a significant interaction effect (exercise × time). *p*‐values above a certain timepoint indicates significant difference between exercised and control samples at the given timepoint. (c) expression of proteins associated with SR Ca^2+^ uptake; SR Ca^2+^ ATPase (SERCA, 1a and 2 to the left and in the middle, respectively) and phospholamban (PLB, to the right). Representative western blots are presented above each associated panel. Numbers 1, 2, and 3 represents 2, 48, and 96 h after exercise, respectively. A standard control sample (ctrl) was added to each run for normalization. (d) representative staining images of SERCA cysteine 674 sulfonylation in control (left) and exercised (right) myofibers. (e) mean SERCA sulfonylation staining intensity in MHC II and I fibers.

We next examined whether reduced force‐generating capacity was linked to impaired myofiber Ca^2+^ homeostasis measured in SR vesicles. SR vesicle Ca^2+^ uptake rate (τ) was negatively affected by exercise and time (*p* = 0.010), with a 30% lower Ca^2+^ uptake rate 96 h after eccentric exercise compared to control (*p* = 0.002; for representative tracing see Figure 3a and results Figure 3b). Uptake rates at 100 and 500 nM [Ca^2+^] normalized to protein content are presented in Figure S1. The observed difference in τ values was not due to changes in the starting [Ca^2+^] at the beginning of the assay, since these values were similar in exercised and control samples (*p* > 0.05).

The reduced SR vesicle Ca^2+^ uptake rate per time unit (τ) led us to investigate whether the impaired Ca^2+^ uptake rate could be ascribed to reduced SERCA activity due to (1) reduced SERCA levels, or (2) altered functional regulation of the protein. Immunoblots (for representative images, see Figure 3c) showed that protein levels of SERCA1, SERCA2, and Phospholamban (PLB), an endogenous inhibitor of SERCA activity, did not change over time and were unaffected by eccentric exercise (Figure 3c). However, our single‐fiber force data indicated a shift in the redox balance towards a more oxidized state. This, together with the results showing impaired Ca^2+^ uptake rate, led us to investigate post‐translational modifications of SERCA, namely sulfonylation at Cysteine 674 (for representative images, see Figure 3d). SERCA sulfonylation staining intensity in both MHC I and MHC II fibers showed an interaction effect between time and exercise (*p* = 0.034 and *p* = 0.023, respectively), with a biphasic pattern of an initial increase in staining intensity 2 h after exercise, before normalizing at 48 h, and a secondary increase 96 h after exercise (Figure 3e). Although there was an interaction effect, post hoc tests revealed no significant differences between exercised and control samples at any specific timepoints. Interestingly, the tendency towards increased staining following exercise was sourced from MHC II fibers.

**FIGURE 4:**
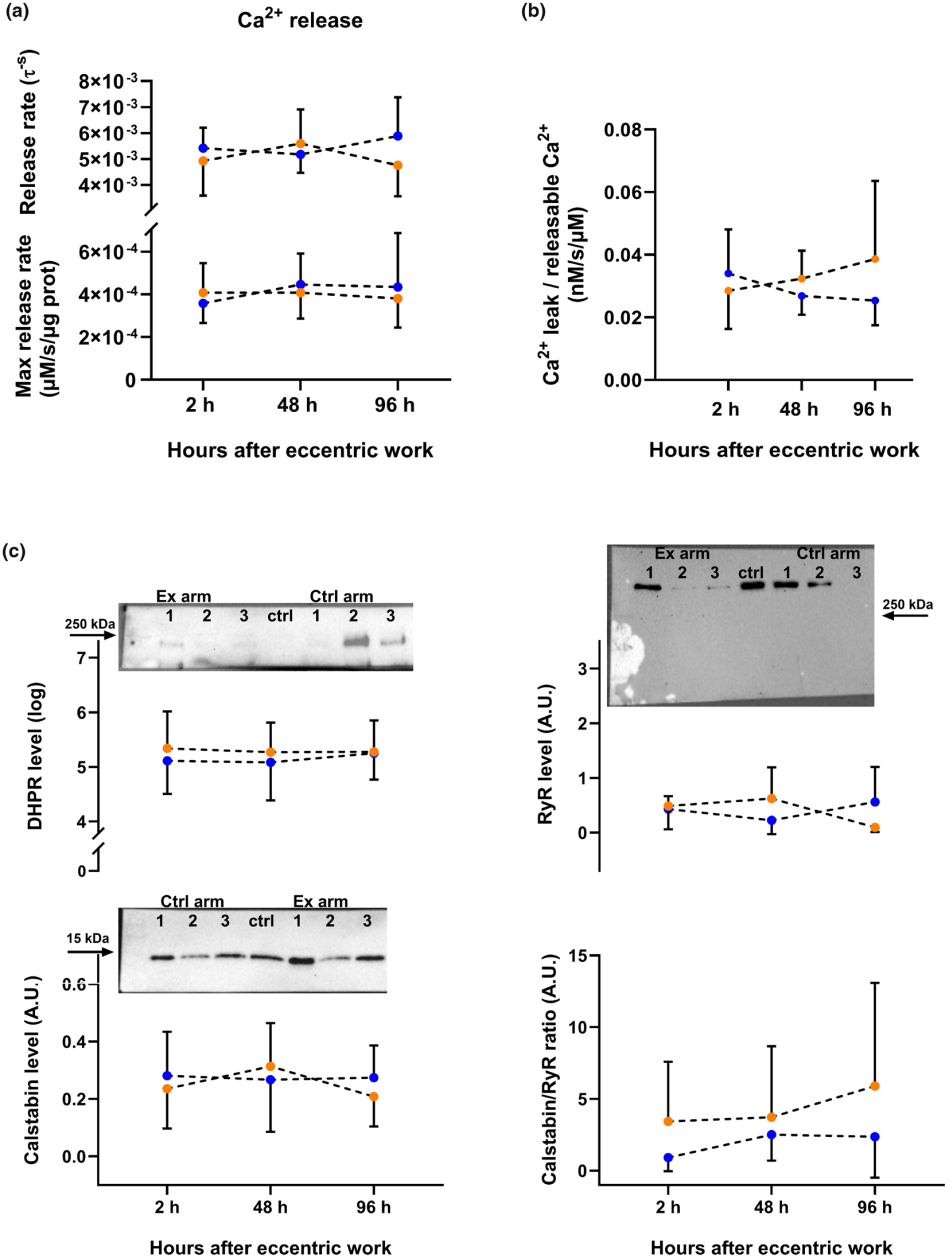
SR vesicle Ca^2+^ release and leak, and expression of proteins associated with Ca^2+^ release and leak. (a) Ca^2+^ release rate (maximal and tau) from SR vesicles. (b) Ca^2+^ leak normalized to SR Ca^2+^ content in SR vesicles. (c) Protein expression of the Ca^2+^ release‐associated proteins, the dihydropyridine receptor (DHPR), the ryanodine receptor (RyR), Calstabin, and the Calstabin/RyR ratio. Representative western blots of DHPR, RyR, Calstabin above each associated panel. Numbers 1, 2, and 3 represents 2, 48, and 96 h after exercise, respectively. A standard control sample (ctrl) was added to each run for normalization. The ctrl sample was not detectable in the DHPR run, hence, the protein expression was not normalized. Orange color represents samples from exercised arm, blue color represents samples from control arm.

Neither SR vesicle Ca^2+^ release rate (maximal and τ, Figure 4a) nor SR vesicle Ca^2+^ leak (Figure 4b) were significantly altered by eccentric exercise. Furthermore, immunoblots (see representative blots in Figure 4c) did not show any change in protein expression levels for RyR (*n* = 10), DHPR (*n* = 12), Calstabin (*n* = 14), or Calstabin/RyR ratio (*n* = 10) (Figure 4c).

### 3.4 Changes in muscle substructure

**FIGURE 5:**
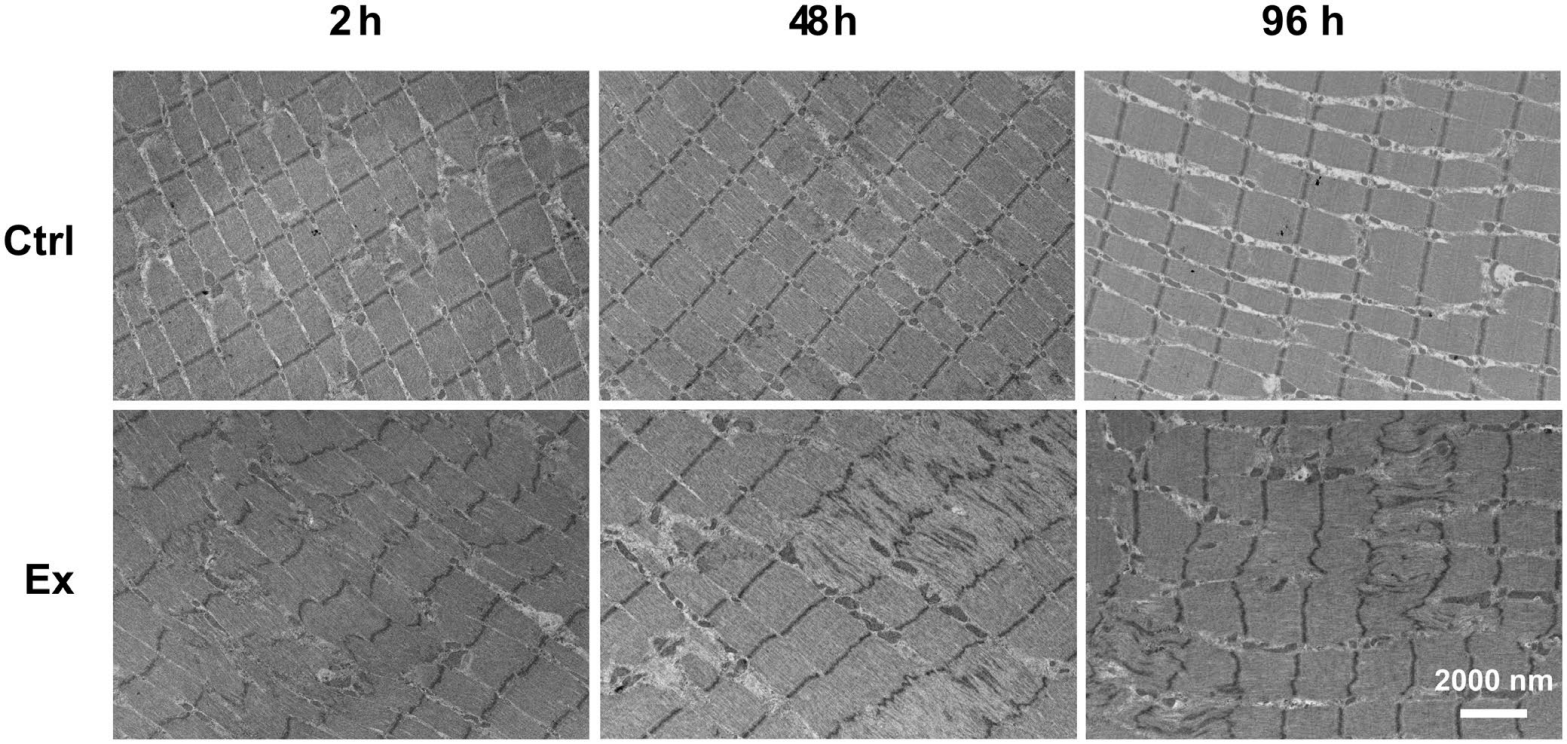
Representative transmission electron micrographs. Micrographs from resting control arm (upper panel) and exercised arm (lower panel) 2, 48, and 96 h after eccentrically, damaging contractions showing disruption of sarcomeres.

Transmission electron microscopy performed on biopsies from the exercised arm confirmed that eccentric exercise induced an expected disruption of myofiber structure. Indeed, representative images revealed characteristic alterations in Z‐discs, including some locations with abnormal zig‐zag patterns and other sites where Z‐discs were destroyed/absent (Figure 5).

**FIGURE 6:**
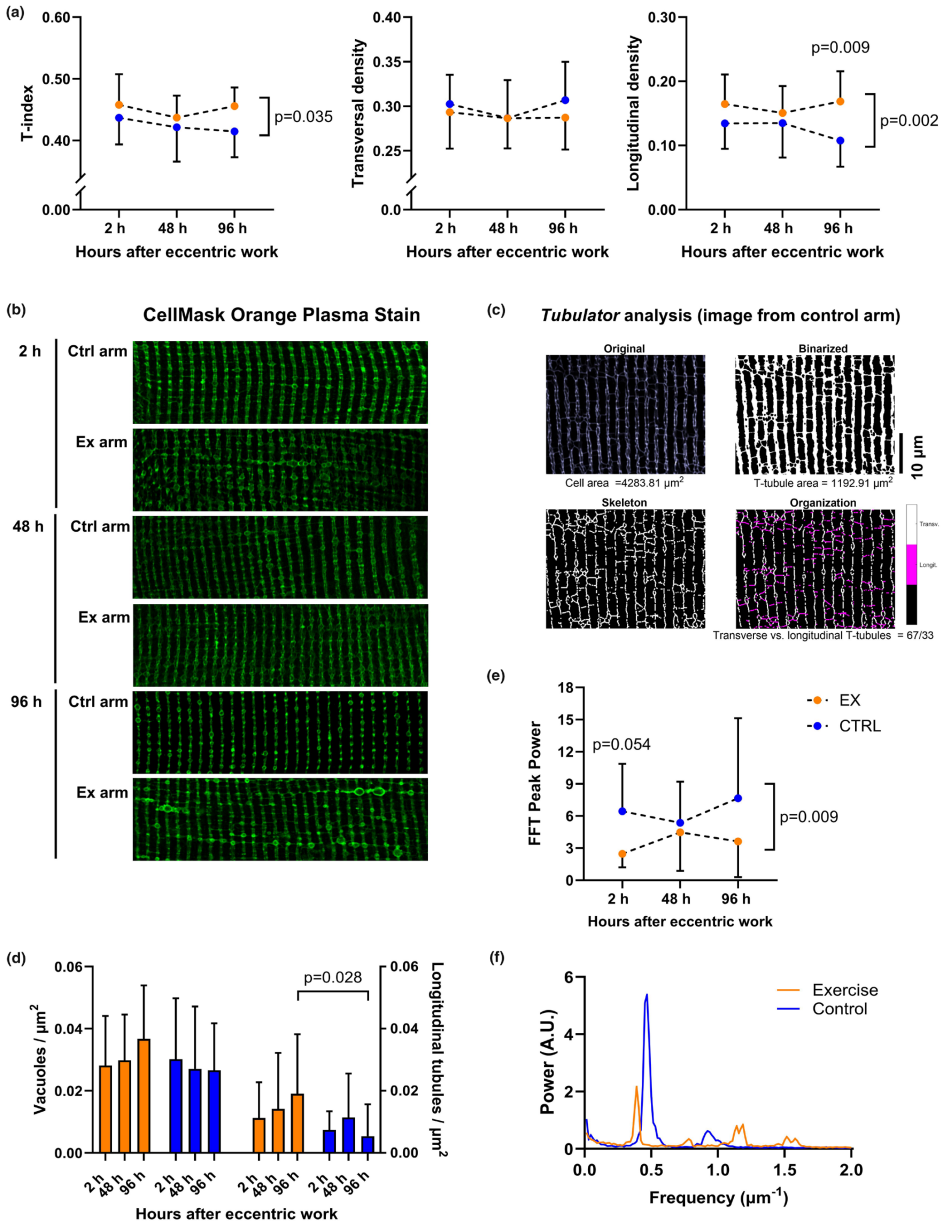
T‐tubular structure after eccentric exercise. (a) Results from *Tubulator*. From left to right: T‐index (the t‐tubular area normalized to whole cell area), density of transversal tubules, and density of longitudinal tubules. Brackets with *p*‐values located on the right side of a figure indicates a significant main effect of exercise. *p*‐values above a certain timepoint indicates significant difference between exercised and control myofibers at the given timepoint. Orange represents results from exercised arm, blue represents results from control arm. The color coding is the same for all plots. (b) Representative images obtained with confocal microscopy of Cell Mask Orange plasma stain in single myofibers. (c) Example of image analysis in *Tubulator*. (d) Manual counting of vacuoles (left *y*‐axis) and longitudinal elements (right *y*‐axis). (e) Results from fast fourier transformation (FFT) depicting peak power from the power spectrum. (f) Representative power spectrum from Fast‐Fourier Transformation (FFT).

We further examined changes in myofiber substructure in greater detail by investigating the t‐system, structures which are critical to Ca^2+^ handling. Here we employed membrane staining (see representative images, Figure 6b) and quantification using *Tubulator* software (see representative images, Figure 6c). These analyses revealed that exercised myofibers exhibited a progressive increase in overall t‐tubule density, the *T‐index*, attributed to augmenting longitudinal density (Figure 6a). Manual counting, ensuring that vacuoles were not misidentified as longitudinal elements, supported the finding of increased longitudinal elements (main effect of exercise, *p* = 0.015) with a 2.8‐fold increase in longitudinal elements 96 h after exercise (*p* = 0.003) and no significant increase in vacuolization (Figure 6d). T‐tubule structural reorganization was further supported by FFT analyses showing an overall decreased peak power, indicating a diminished presence of transverse elements in the exercised myofibers (*p* = 0.009, Figure 6, representative tracing in panel f and results in panel e).

Determination of single‐fiber MHC from the t‐tubule imaging revealed that 90 fibers predominantly expressed MHC II, 2 were hybrid fibers, and 15 primarily expressed MHC I fibers. Due to the low number of MHC I‐fibers, subgroup analysis was only performed for MHC II fibers. Results did not differ from the analysis of the whole sample, but significant changes became tendencies because of the lower sample size. Consequently, the results for the whole sample were used for further data interpretation and are presented here.

## 4 DISCUSSION

The eccentric exercise protocol caused a 50% decline in maximal force‐generating capacity which did not fully recover during the following 4 days. The reduced force‐generating capacity was accompanied by a reduction in electrically evoked force, especially at low frequencies as evident by the 20/50 Hz force ratio. Next, we examined single‐fiber contractile function to see whether the whole‐muscle force impairments could be attributed to alterations in the contractile apparatus and whether t‐system and SR vesicle Ca^2+^ uptake and release rates were affected.

### 4.1 Alterations in contractile apparatus function

Single fiber contractile function was investigated using exposure to increasing [Ca^2+^] in samples collected 48 h after damaging exercise. This timepoint was selected based on previous literature showing that 48 h is the breaking point where some fibers recover while others undergo necrosis (Paulsen et al., 2012). Hence, this study might shed light on individual fiber differences leading to the two very different outcomes. In line with the *in vivo* results, contractile apparatus specific force (P_0_) was significantly decreased at this timepoint in exercised MHC I‐fibers. This contractile impairment likely contributes to the force deficit observed *in vivo*. Reduced specific force suggests that the contractile apparatus is altered and/or damaged. Indeed, this was confirmed by the structural disruption and sarcomere smearing as evidenced by electron micrographs.

Surprisingly, P_0_ did not change after the damaging exercise in MHC II‐fibers. We also did not observe exercise‐related changes in the steepness of the force‐Ca^2+^ relationship, that is, Hill coefficient, indicating that other factors must contribute to the exercise‐induced functional impairment of the contractile apparatus. However, a previous study of regenerative EDL fibers (MHC II‐fibers) which examined a 7‐day timepoint following myotoxic injury reported a reduced Hill coefficient, which resembled the force‐Ca^2+^ relationship of soleus fibers (MHC I‐fibers) (Gregorevic et al., 2004). Notably, the myotoxic injury model induces more extensive injury than exercise‐induced injury. Based on the lack of exercise‐induced change in the Hill coefficient in our study, it seems likely that the fibers studied were not regenerative at the timepoint examined.

Myofiber structural damage was indicated by the large decline in whole muscle force‐generating capacity and further confirmed in electron micrographs (Figure 5). Severe structural damage would lead to reduced P_0_. Studies have shown that MHC II‐fibers are more prone to suffer muscle damage (Choi, 2014; Schiaffino & Reggiani, 2011), and we therefore hypothesized that MHC II‐fibers would show impaired P_0_. Notably, myofiber imaging showed that the damage is not evenly distributed, meaning that healthy and severely damaged fibers are located next to each other (Fridén & Lieber, 1998). Based on this, we suspect that the MHC II‐fiber P_0_ might be affected by a selection bias during the isolation of fibers, where large, seemingly vital fibers could have been selected over smaller and/or less vital‐looking fibers. Consequently, the lack of exercise‐induced changes in MHC II‐fiber P_0_ reported here should be interpreted with caution.

Interestingly, we observed an increase in Ca^2+^ sensitivity after eccentric exercise in MHC II‐fibers only. This increase was subsequently reversed to untreated, control levels by a strong reducing agent, suggesting oxidation‐induced alterations of the contractile apparatus affect the Ca^2+^ sensitivity. This is in line with observations of an increased myofibrillar Ca^2+^ sensitivity of fast‐twitch fibers in the early stage of prolonged low‐frequency fatigue of rat muscle (Watanabe et al., 2015). ROS/RNS exposure to myofibers has been shown to both increase Ca^2+^ sensitivity with no increase in maximum force and to decrease Ca^2+^ sensitivity and decrease maximum force, depending on the duration of the exposure, the type of oxidant, and the oxidant concentration. Longer exposure showed negative effects, while shorter exposure showed positive effects, specifically in regards to Ca^2+^ sensitivity (Andrade et al., 1998b; Lamb & Posterino, 2003; Lamb & Westerblad, 2011). Further, Mollica et al. (2012) proposed a possible mechanism for the ROS‐induced MHC II‐fiber increase in Ca^2+^ sensitivity: S‐glutathionylation. S‐glutathionylation of troponin I may protect molecules from oxidative stress while also increasing Ca^2+^ sensitivity of the contractile apparatus with little or no change in maximal force (Mollica et al., 2012; Watanabe et al., 2015). S‐glutathionylation is limited to MHC II fibers and has been shown to occur in exercising human muscle (Mollica et al., 2012). This phenomenon is also reversible, which aligns well with our results showing an antioxidant‐dependent decrease in Ca^2+^ sensitivity in MHC II‐fibers, without any exercise‐dependent change in maximal force. It should be noted that subjecting skinned fibers to repeated force‐pCa trials can result in a progressive decrease in Ca^2+^ sensitivity, and that this effect is estimated to be ~−0.012 pCa units (Lamb & Posterino, 2003; Mollica et al., 2012). Importantly, our results show a change in Ca^2+^ sensitivity of −0.1 pCa units, which is well below the systematic error of repeated trials.

To summarize, we observed a large decline in *in vivo* force development following eccentric exercise, a finding which was linked to impaired contractile function in MHC I‐fibers, but surprisingly not in the MHC II‐fibers analysed. However, we suspect that a selection bias influenced the MHC II‐results. Interestingly, the contractile apparatus showed increased Ca^2+^ sensitivity, probably related to reversible oxidation, and decreased P_0_ when treating MHC II‐fibers with a reducing agent. Therefore, the oxidation 48 h after eccentric exercise is likely modest and hence, not negatively affecting contractile function. We and others (Mollica et al., 2012; Watanabe et al., 2015) speculate that the increased Ca^2+^ sensitivity of the contractile apparatus possibly is a compensating mechanism to attenuate the force deficit associated with fatiguing or damaging exercise.

### 4.2 Impaired myofiber Ca^2+^ handling

In addition to direct changes in the contractile apparatus, changes in myofiber Ca^2+^ handling, that is, SR and the t‐system may impact contractile function after muscle‐damaging exercise.

#### 4.2.1 T‐tubular structure

T‐tubular structure showed a time‐dependent formation of longitudinal elements, which led to an overall increase in t‐tubule density, and a loss of structural regularity (lower peak power in FFT analysis) was observed. Similar alterations were previously observed in both fast‐ and slow‐twitch fibers following downhill running in rats (Takekura et al., 2001) and after heavy‐load resistance exercise in humans (Cully et al., 2017). In the rat study, an immediate increase in longitudinal tubules was observed after exercise, and a secondary increase followed a few days later. Although differing sampling time resolutions make direct comparisons challenging, a similar trend was found in this study and in Cully et al. (2017). A possible explanation for increased longitudinal tubules relates to Ca^2+^ overload and handling. Eccentrically, damaging exercise has previously been reported to disrupt Ca^2+^ homeostasis, including an increase in resting, intracellular [Ca^2+^] that is detrimental for myofiber function (Figure 2, Allen et al., 2010). Interestingly, it has been proposed that acute Ca^2+^ overload can result in local blocking of Ca^2+^ release as a protective mechanism, while also promoting long‐term fatigue (Lamb et al., 1995). Therefore, increased t‐tubule area may be a protective mechanism that allows augmented Ca^2+^ extrusion from the cytosol. Indeed, the t‐tubular network has been proposed to handle excessive intracellular [Ca^2+^] by sequestering Ca^2+^ in vacuoles, and a previous study showed that heavy resistance exercise caused increased vacuolization (Cully et al., 2017). Although we did not observe increased vacuolization, the overall t‐system area and amount of longitudinal tubules were increased, indicating that the tubular network expanded due to the damaging exercise. Takekura et al. (2001) and Cully et al. (2017) also speculated that longitudinal tubule formation is related to sarcomere disruption following exercise‐induced muscle damage. This aligns well with our data from transmission electron micrographs (Figure 5) and previous observations after a similar exercise protocol where sarcomeres indeed were disrupted (Lauritzen et al., 2009). We speculate that cytosolic Ca^2+^ influx due to membrane disruptions in both plasmalemma and SR is related to sarcomere disruptions, and that both vacuolization and increased longitudinal tubules could be a mechanism to cope with the accumulation of intracellular Ca^2+^. In support of this, a SERCA 2 knock‐out model (Swift et al., 2012), exhibited increased longitudinal tubules, maintained transverse tubules and a resulting overall increase in t‐tubule density. The authors speculated that the longitudinal tubule growth might be a compensatory mechanism to allow for increases in Ca^2+^ fluxes through the plasmalemmal Na^+^/Ca^2+^ exchanger, and this might also be true after damaging exercise. Taken together, our results, together with existing literature, indicate that the structural t‐tubule changes observed are related to local cytosolic Ca^2+^ overload.

#### 4.2.2 SR vesicle Ca^2+^ handling

Cytosolic Ca^2+^ overload is a likely result of exercise‐induced muscle damage and may be linked to the generation of both secondary damage and depressed force‐generating capacity. To delve deeper into this issue, we investigated SR vesicle Ca^2+^ handling and found a time‐ and exercise‐dependent decrease in SR vesicle Ca^2+^ uptake rate. Interestingly, while the exercised muscle showed significantly reduced SR vesicle Ca^2+^ uptake rates 96 h after exercise, SR vesicle Ca^2+^ release and leak were not affected by the eccentric, damaging exercise. Others have reported both decreased Ca^2+^ uptake and release (Ingalls et al., 1998) and no change in Ca^2+^ uptake and release (Nielsen et al., 2007) after eccentric exercise. Notably, Nielsen and co‐workers (Nielsen et al., 2007) utilized soleus muscle from rats, which consists of mainly MHC I‐fibers. In contrast, our SR vesicle preparation originated from the human m. biceps brachii, where we quantified by muscle cross sections that the m. biceps brachii consisted of 51 ± 6% MHC II fibers. Moreover, the decreased SR Ca^2+^ release and uptake rates reported by Ingalls and co‐workers were observed in mice EDL, which consist of 99% MHC II fibers (Crow & Kushmerick, 1982). Furthermore, MHC II‐fibers have been shown to be more prone to muscle damage (Choi, 2014; Schiaffino & Reggiani, 2011). We therefore speculate that reduced Ca^2+^ release in MHC II‐fibers likely is a consequence of maximal eccentric contractions, but that the substantial presence of MHC I‐fibers in our material could mask this finding. Conversely, only MHC I fibers showed impaired maximal Ca^2+^‐activated force, suggesting that the contractile apparatus force‐generating capacity of these fibers was negatively affected by eccentric exercise. However, as mentioned above, we suspect that there may have been a selection bias in the MHC II‐fiber analysis. This bias will not have occurred in the SR vesicle assay since this analysis is based on a muscle homogenate. The reported, extensive, force decline in the study by Ingalls et al. (1998) was of longer duration than that which we presently observed (−50% at day 5 post‐injury vs. −23% at day 4 post‐injury). This indicates that the EDL exercise protocol most likely induced greater mechanical overload and damage, which is expected since they performed 150 electrically induced eccentric actions compared to our 50 voluntary eccentric actions. Together with the different fiber type distribution, this discrepancy likely explains the differences observed.

Importantly, SR vesicle Ca^2+^ handling did not show any exercise‐related changes in the early‐ and mid‐timepoints (2 and 48 h, respectively) after exercise. This suggests that neither the RyR function or protein level nor the SERCA function or protein level is altered in the early phase. This is also supported by the data from the immunoblotting and from a previous study (Ingalls et al., 1998). Alternatively, SERCA could be subject to both stimulating and inhibitory processes such as cytosolic Ca^2+^ overload and increased ROS/RNS simultaneously, resulting in an overall unchanged Ca^2+^ uptake rate.

We cannot exclude that physiological Ca^2+^ release and ‐leak are altered since (1) the DHPR‐RyR interplay is not considered with the SR vesicle assay, and (2) the method assesses protein function in an *in vitro* vesicle preparation. No immunoprecipitation of the SR vesicles has been performed to investigate protein association present, such as the RyR‐Calstabin complex, which is an important regulator of physiological SR Ca^2+^ leak (Brillantes et al., 1994). Furthermore, the SR vesicle assay cannot distinguish the source of the Ca^2+^ leak. The leak could also theoretically occur across the membrane if vesicles were damaged. However, this seems unlikely as such damage would inhibit the filling of the vesicles with Ca^2+^ by SERCA.

Our results show that the 20/50 Hz force ratio 1 h after exercise was decreased, indicative of low‐frequency fatigue, and previous work has indicated that impaired Ca^2+^ release is the major cause of low‐frequency fatigue (Watanabe et al., 2015; Westerblad et al., 1993). After stretched contractions, single‐fiber studies using caffeine to elicit force generation have also shown that reduced Ca^2+^ release (and reduced Ca^2+^ sensitivity) contribute to the reduced force‐generating capacity (Balnave & Allen, 1995). The study by Ingalls et al. (1998) also show that EC‐uncoupling accounts for the majority of the force loss in the first days after damaging exercise. In fact, the DHPR‐RyR interplay has been suggested to be the site of the EC coupling defect (Ingalls et al., 1998), and this is in line with no change in SR vesicle Ca^2+^ uptake since only RyRs are responsible for Ca^2+^ release from the SR vesicles. The early phase force loss (5 min after exercise) in the current study is likely caused by structural disruption of sarcomere integrity (as evident in electron micrographs), modification to or proteolytic degradation of proteins, and metabolic fatigue (Lamb, 2002), all of which affect EC‐coupling, and possibly through DHPR‐RyR Ca^2+^ release.

The observed reduction in SR vesicle Ca^2+^ uptake rate 96 h after exercise is likely contributing to the impairment in maximal force‐generating capacity evident at the same time. Maximal force generation requires trains of high‐frequency action potentials usually above 50 Hz (Desmedt & Godaux, 1977). The amount of Ca^2+^ released by a single action potential is almost constant over a wide range of SR Ca^2+^ content (Posterino & Lamb, 2003). Fast refilling is therefore required to sustain consistent Ca^2+^ release during repeated action potentials. Otherwise, SR may eventually deplete, leading to decreasing Ca^2+^ release and an accompanying further decline in maximal force production (Posterino & Lamb, 2003).

With our data showing impaired SR vesicle Ca^2+^ uptake rate 96 h after exercise, we hypothesized that it could be due to either a decreased SERCA protein level or impaired SERCA function. We did, however, not observe any changes in protein levels of SERCA1 or SERCA2, evident by immunoblotting, so we further investigated if function could be impaired by changes in PLB levels. PLB inhibits SERCA by decreasing its Ca^2+^ affinity, and phosphorylation of PLB partially reverses the inhibition (Akin et al., 2013). We did not detect any changes in PLB protein expression. Unfortunately, our samples were not appropriately prepared for analysis of phosphorylation status (i.e., no phosphatase inhibitor was added to the homogenization buffer). Therefore, analysis of phosphorylation status could not be conducted. Due to the contractile apparatus showing indications of altered redox status and altered redox status may affect Ca^2+^ uptake (Andrade et al., 1998a; Xu et al., 1997), another possible explanation for reduced Ca^2+^ uptake emerged: post‐translational modification of SERCA, such as oxidation of specific SERCA residues.

#### 4.2.3 SERCA sulfonylation

Several SERCA cysteine residues are indeed sensitive to oxidation (Viner et al., 2000), with Cys674 and −675 having been shown to be particularly sensitive to age‐dependent oxidation *in vivo*, as well as peroxinitrate oxidation. SERCA Cys674 and −675 show that, when exposed to H_2_O_2_, the reaction product is sulfenic acid, and exposure of higher levels of peroxides results in increasing sulfonylation (Dremina et al., 2007). A recent study further show that eccentric contractions produce more H_2_O_2_ than concentric contraction, and the increase is sustained for at least 1 h after eccentric contractions (Kano et al., 2024). Our data show that time and exercise significantly affected SERCA Cys 674 sulfonylation with increases at 2 h, before normalization at 48 h, and a secondary increase at 96 h after exercise. However, neither of the specific timepoints were significantly different between exercise and control muscle in the post‐hoc analyses. Other cysteine residues than Cys 674 can also be target for oxidation (Dremina et al., 2007). For example, Dremina and colleagues highlighted that oxidation of a single cysteine residue might not explain impaired SERCA function and that impaired SERCA function could represent the sum of all oxidized cysteine residues.

In the pooled data for SERCA sulfonylation and particularly in MHC II‐fibers, we observed a biphasic pattern with an immediate, albeit non‐significant, increase in sulfonylation 2 h after exercise. This increase was followed by a return to control levels at 48 h, before a second, smaller rise in sulfonylation 96 h after exercise. This biphasic pattern corresponds well with the current knowledge about ROS, exercise, and EIMD. The first phase being the acute exercise‐induced ROS accumulation, likely caused by both increased oxidative phosphorylation due to exercise (Michelucci et al., 2021) and Ca^2+^ overload (Görlach et al., 2015) possibly due to membrane disruptions (McNeil & Khakee, 1992). Immediate extracellular neutrophil accumulation may also contribute to the first phase of ROS generation (McLoughlin et al., 2003; Paulsen, Crameri, et al., 2010). Although a non‐significant increase in SERCA Cys 674 sulfonylation 2 h after exercise was observed, the SR vesicle Ca^2+^ uptake rate was unchanged at this timepoint. As pointed out previously, SERCA can be regulated by post‐translational modifications directly and by other regulatory proteins such as PLN, but also by cytosolic Ca^2+^. Exposure to ROS/RNS can in fact both stimulate and inhibit SERCA (Xu & Van Remmen, 2021), and the same is true for exposure to free Ca^2+^. Theoretically, Ca^2+^ uptake could be unchanged if inhibition of SERCA by sulfonylation and stimulation of SERCA by increased cytosolic [Ca^2+^] occurred simultaneously.

The second phase (from 48 to 96 h after exercise) involves the invasion of ROS‐generating inflammatory cells into the injured tissue several days after the damaging exercise (Paulsen, Crameri, et al., 2010; Paulsen, Egner, et al., 2010; Zerba et al., 1990). This phase might also involve an overload of cytosolic Ca^2+^ due to membrane disruptions relating to necrosis, which potentially further increase ROS production. In the second phase, impaired SR Ca^2+^ uptake can also contribute to further Ca^2+^ overload. Consequently, we suggest that the damage‐induced accumulation of ROS‐producing inflammatory cells, as well as the necrosis‐related membrane disruptions which causes a second Ca^2+^ overload and further increased ROS production, is a likely explanation for the impaired SR vesicle Ca^2+^ uptake rate 96 h after exercise.

## 5 CONCLUSION

Our employed eccentric exercise protocol induced an expected severe and prolonged force decline, and we confirmed that there was accompanying muscle damage. We investigated possible underlying mechanisms relating to Ca^2+^ handling and Ca^2+^ sensitivity. As hypothesized, we observed t‐tubule remodeling and impaired SR vesicle Ca^2+^ uptake 96 h after damaging exercise. Single‐fiber force measurements, somewhat surprisingly, showed no exercise‐dependent change in maximal specific force in MHC II‐fibers, but substantial reductions in MHC I‐fibers. Furthermore, the single fiber force‐Ca relationship before and after antioxidant treatment indicated a shift in redox status in damaged muscle with elevated Ca^2+^ sensitivity and a tendency towards increased oxidation. Importantly, a time‐dependent biphasic rise in SERCA sulfonylation was observed, suggesting that increased oxidation may explain reduced SR vesicle Ca^2+^ uptake through impaired SERCA function. In conclusion, SR vesicle Ca^2+^ uptake was impaired 96 h after damaging exercise, likely due to increased oxidative stress. The tubular network showed structural changes, with increased longitudinal elements at the same timepoint, and we speculate that this is a compensatory mechanism aimed at reducing intracellular Ca^2+^ concentration.

## AUTHOR CONTRIBUTIONS

Experiments were carried out at the Department of Physical Performance, Norwegian School of Sport Sciences, Institute of Experimental Medical Research, Oslo University Hospital, and Department of Sports Science and Clinical Biomechanics, University of Southern Denmark. VH, PKL, MF, OS, NØ, WEL, GP, and TR contributed to conception of the work. VH, PKL, MF, OS, NØ, WEL, GP, and TR contributed to develop methods and/or performing experiments. VH, PKL, MF, OS, NØ, WEL, GP, and TR contributed to drafting the work and/or revising it critically. All authors have approved the final version of the manuscript. All authors agree to be accountable for all aspects of the work. All authors agree that the current list of authors is justified and that all those who qualify for authorship are listed.

## FUNDING INFORMATION

The present study was not supported by an external source. The study was internally funded by the Department of Physical Performance at the Norwegian School of Sport Sciences.

## CONFLICT OF INTEREST STATEMENT

The authors declare no conflict of interest.

## Supporting information


Figure S1.

## Data Availability

All the data in the present study are available from the corresponding author upon request.
