# Supplementary material for: Myofiber structure, sarcoplasmic reticulum Ca2+ handling, and contractile function after muscle‐damaging exercise in humans
Source: Physiol Rep. 2025 Feb 2;13(3):e70204. doi: 10.14814/phy2.70204 (PMC11788311; doi:10.14814/phy2.70204)
Supplement: Supplementary file 1 — Figure S1. [file PHY2-13-e70204-s001.docx]

RESEARCH ARTICLE

running head: Myofiber structure and Ca^2+^ handling after damaging exercise

Myofiber structure, Sarcoplasmic reticulum Ca^2+^ handling, and contractile function after muscle damaging exercise in humans

Supplementary

Figure 1. Ca^2+^ uptake rates at 500 mM (panel A) and 100 nM [Ca^2+^] (panel B) normalized to protein content. Orange color represents samples from exercised arm, blue color represents samples from control arm.
